# Supplementary material for: Repurposing the yellow fever vaccine for intratumoral immunotherapy
Source: EMBO Mol Med. 2019 Nov 19;12(1):e10375. doi: 10.15252/emmm.201910375 (PMC6949490; doi:10.15252/emmm.201910375)
Supplement: Supplementary file 1 — Appendix [file EMMM-12-e10375-s001.pdf]

APPENDIX INFORMATION

Repurposing the Yellow Fever Vaccine for intratumoral Immunotherapy

M. Angela Aznar, Carmen Molina, Alvaro Teijeira, Inmaculada Rodriguez, Arantza Azpilikueta, Saray Garasa, Alfonso R. Sanchez-Paulete, Luna Cordeiro, Iñaki Etxeberria, Maite Alvarez, Sergio Rius-Rocabert, Estanislao Nistal-Villan, Pedro Berraondo, Ignacio Melero

APPENDIX MATERIAL

Appendix Table S1

Appendix Table S1. Exact p values

Figure	Comparison	p-values	Statistical test
**1E**	tumor growth	<0.0001	non linear regresion curve fit, extra sum-of-squares F tests
	survival	0.086	Log-rank (Mantel-Cox) test
**1G**	tumor growth	<0.0001	non linear regresion curve fit, extra sum-of-squares F tests
	survival	0.0018	Log-rank (Mantel-Cox) test
**1I**	tumor growth	<0.0001	non linear regresion curve fit, extra sum-of-squares F tests
**1J**	tumor growth, day 21	0.0964	non linear regresion curve fit, extra sum-of-squares F tests
	tumor growth, day 17	0.0014	non linear regresion curve fit, extra sum-of-squares F tests
	tumor growth, day 13	<0.0001	non linear regresion curve fit, extra sum-of-squares F tests
**2B**	Control+RIgG vs 17D+RIgG	0.0019	non linear regresion curve fit, extra sum-of-squares F tests
	17D+RIgG vs 17D+αCD8	<0.0001	non linear regresion curve fit, extra sum-of-squares F tests
	17D+RIgG vs 17D+αCD4	0.9088	non linear regresion curve fit, extra sum-of-squares F tests
	Control+RIgG vs 17D+RIgG	<0.0001	non linear regresion curve fit, extra sum-of-squares F tests
	17D+RIgG vs 17D+αCD8	<0.0001	non linear regresion curve fit, extra sum-of-squares F tests
**2C**	17D+RIgG vs 17D+αCD4	0.0007	non linear regresion curve fit, extra sum-of-squares F tests
	Control+RIgG vs 17D+RIgG	0.0776	non linear regresion curve fit, extra sum-of-squares F tests

	Control+RIgG vs 17D+αCD8	0.6042	non linear regresion curve fit, extra sum-of-squares F tests
	Control+RIgG vs 17D+αCD4	0.084	non linear regresion curve fit, extra sum-of-squares F tests
	17D+RIgG vs 17D+αCD8	0.0143	non linear regresion curve fit, extra sum-of-squares F tests
	17D+RIgG vs 17D+αCD4	0.0215	non linear regresion curve fit, extra sum-of-squares F tests
**2D**	17D+RIgG vs 17D+αCD4	0.2545	Log-rank (Mantel-Cox) test
	17D+αCD8 vs 17D+αCD4	0.027	Log-rank (Mantel-Cox) test
	Control+RIgG vs 17D+RIgG	0.0776	Log-rank (Mantel-Cox) test
	Control+RIgG vs 17D+αCD8	0.437	Log-rank (Mantel-Cox) test
	Control+RIgG vs 17D+αCD4	0.084	Log-rank (Mantel-Cox) test
	17D+RIgG vs 17D+αCD8	0.0143	Log-rank (Mantel-Cox) test
**3B**	Control+αCD137 vs 17D+αCD137	<0.0001	non linear regresion curve fit, extra sum-of-squares F tests
	Control+αPD-1 vs 17D+αPD-1	0.0084	non linear regresion curve fit, extra sum-of-squares F tests
	17D+αCD137 vs 17D+RIgG	<0.0001	non linear regresion curve fit, extra sum-of-squares F tests
	17D+αPD-1 vs 17D+RIgG	<0.0001	non linear regresion curve fit, extra sum-of-squares F tests
	17D+αCD137 vs Control+RIgG	<0.0001	non linear regresion curve fit, extra sum-of-squares F tests
	Control+RIgG vs 17D+RIgG	0.6175	non linear regresion curve fit, extra sum-of-squares F tests
	17D+αPD-1 vs Control+RIgG	<0.0001	non linear regresion curve fit, extra sum-of-squares F tests
**3C**	Control+αCD137 vs 17D+αCD137	0.0952	non linear regresion curve fit, extra sum-of-squares F tests

	Control+αPD-1 vs 17D+αPD-1	0.0005	non linear regresion curve fit, extra sum-of-squares F tests
	17D+αCD137 vs 17D+RIgG	<0.0001	non linear regresion curve fit, extra sum-of-squares F tests
	17D+αPD-1 vs 17D+RIgG	<0.0001	non linear regresion curve fit, extra sum-of-squares F tests
	17D+αCD137 vs Control+RIgG	<0.0001	non linear regresion curve fit, extra sum-of-squares F tests
	Control+RIgG vs 17D+RIgG	0.7917	non linear regresion curve fit, extra sum-of-squares F tests
	17D+αPD-1 vs Control+RIgG	<0.0001	non linear regresion curve fit, extra sum-of-squares F tests
**3D**	17D+αCD137 vs Control+αCD137	0.306	Log-rank (Mantel-Cox) test
	17D+αPD-1 vs Control+αPD-1	0.9666	Log-rank (Mantel-Cox) test
	17D+αPD-1 vsControl+RIgG	0.07988	Log-rank (Mantel-Cox) test
	17D+αCD137 vs Control+RIgG	0.0005	Log-rank (Mantel-Cox) test
	17D+αPD-1 vs 17D+RIgG	0.6548	Log-rank (Mantel-Cox) test
	17D+αCD137 vs 17D+RIgG	0.0005	Log-rank (Mantel-Cox) test
	17D+RIgG vs Control+RIgG	0.7775	Log-rank (Mantel-Cox) test
	17D+αCD137 Control+αPD-1	0.0007	Log-rank (Mantel-Cox) test
	Control+αCD137 vs Control+RIgG	0.0572	Log-rank (Mantel-Cox) test
	Control+αPD-1 vs Control+αCD137	0.101	Log-rank (Mantel-Cox) test
**4B**	WT 17D+RIgG vs WT Control+RIgG	0.0027	non linear regresion curve fit, extra sum-of-squares F tests
	KO 17D+RIgG vs KO Control+RIgG	<0.0001	non linear regresion curve fit, extra sum-of-squares F tests
	WT 17D+RIgG vs KO 17D+RIgG	0.2885	non linear regresion curve fit, extra sum-of-squares F tests
	WT Control+RIgG vs KO Control+RIgG	0.089	non linear regresion curve fit, extra sum-of-squares F tests

	WT 17D+αCD137 vs WT Control+αCD137	0.0006	non linear regresion curve fit, extra sum-of-squares F tests
	KO 17D+αCD137 vs KO Control+αCD137	0.0009	non linear regresion curve fit, extra sum-of-squares F tests
	WT 17D+αCD137 vs KO 17D+αCD137	<0.0001	non linear regresion curve fit, extra sum-of-squares F tests
	WT Control+αCD137 vs KO Control+αCD137	<0.0001	non linear regresion curve fit, extra sum-of-squares F tests
	WT 17D+αPD-1 vs WT Control+αPD-1	0.0199	non linear regresion curve fit, extra sum-of-squares F tests
	KO 17D+αPD-1 vs KO Control+αPD-1	0.976	non linear regresion curve fit, extra sum-of-squares F tests
	WT 17D+αPD-1 vs KO 17D+αPD-1	0.0011	non linear regresion curve fit, extra sum-of-squares F tests
	WT Control+αPD-1 vs KO Control+αPD-1	<0.0001	non linear regresion curve fit, extra sum-of-squares F tests
**4C**	WT 17D+RIgG vs WT Control+RIgG	0.0164	non linear regresion curve fit, extra sum-of-squares F tests
	KO 17D+RIgG vs KO Control+RIgG	0.012	non linear regresion curve fit, extra sum-of-squares F tests
	WT 17D+RIgG vs KO 17D+RIgG	0.0042	non linear regresion curve fit, extra sum-of-squares F tests
	WT Control+RIgG vs KO Control+RIgG	<0.0001	non linear regresion curve fit, extra sum-of-squares F tests
	WT 17D+αCD137 vs WT Control+αCD137	0.5086	non linear regresion curve fit, extra sum-of-squares F tests
	KO 17D+αCD137 vs KO Control+αCD137	<0.0001	non linear regresion curve fit, extra sum-of-squares F tests
	WT 17D+αCD137 vs KO 17D+αCD137	0.0382	non linear regresion curve fit, extra sum-of-squares F tests
	WT Control+αCD137 vs KO Control+αCD137	<0.0001	non linear regresion curve fit, extra sum-of-squares F tests

	WT 17D+αPD-1 vs WT Control+αPD-1	0.0067	non linear regresion curve fit, extra sum-of-squares F tests
	KO 17D+αPD-1 vs KO Control+αPD-1	0.0011	non linear regresion curve fit, extra sum-of-squares F tests
	WT 17D+αPD-1 vs KO 17D+αPD-1	<0.0001	non linear regresion curve fit, extra sum-of-squares F tests
	WT Control+αPD-1 vs KO Control+αPD-1	<0.0001	non linear regresion curve fit, extra sum-of-squares F tests
**5B**	%CD8/CD45+	0.3112	Mann-Whithey test, Two-tailed
	CD8+ absol/gram	0.0221	Mann-Whithey test, Two-tailed
**5C**	%CD25+FOXP3+/CD45+	0.0023	Mann-Whithey test, Two-tailed
	CD25+FOXP3+ absol/gram	0.051	Mann-Whithey test, Two-tailed
**5D**	%CD8+/Treg	0.0012	Mann-Whithey test, Two-tailed
	Tconv/Treg	0.0023	Mann-Whithey test, Two-tailed
**5E**	%NK1.1/CD45+	0.0012	Mann-Whithey test, Two-tailed
	NK1.1+ absol/gram	0.5338	Mann-Whithey test, Two-tailed
**5F**	CD137 MFI in CD4	0.0140	Mann-Whithey test, Two-tailed
	CTLA-4 MFI in CD4	0.0169	Mann-Whithey test, Two-tailed
	PD-1 MFI in CD4	0.0221	Mann-Whithey test, Two-tailed
	CD137 MFI in CD8	0.5338	Mann-Whithey test, Two-tailed
	CTLA-4 MFI in CD8	0.0169	Mann-Whithey test, Two-tailed
	PD-1 MFI in CD8	0.0082	Mann-Whithey test, Two-tailed
**5G**	%CD8/CD45+	0.8357	Mann-Whithey test, Two-tailed
	CD8+ absol/gram	0.0221	Mann-Whithey test, Two-tailed
	CD137+CD8+ absol/gram	0.0082	Mann-Whithey test, Two-tailed
	PD-1+CD8+ absol/gram	0.0221	Mann-Whithey test, Two-tailed
	%NK1.1/CD45+	0.1375	Mann-Whithey test, Two-tailed
	NK1.1 absol/gram	0.0082	Mann-Whithey test, Two-tailed

	CD137+NK	0.0047	Mann-Whithey test, Two-tailed
**6B**	Immunized vs. Naive	0.0249	Kruskal-Wallis test + Dunn's multiple comparisons correction
	Immunized vs. C+	0.0089	Kruskal-Wallis test + Dunn's multiple comparisons correction
	Naive vs. C+	>0.9999	Kruskal-Wallis test + Dunn's multiple comparisons correction
**6C**	17D Immunized vs. 17D Naive	<0.0001	non linear regresion curve fit, extra sum-of-squares F tests
	Control Immunized vs. 17D Naive	0.571	non linear regresion curve fit, extra sum-of-squares F tests
	Control Immunized vs. Control Naive	0.0084	non linear regresion curve fit, extra sum-of-squares F tests
	17D Immunized vs. Control Immunized	<0.0001	non linear regresion curve fit, extra sum-of-squares F tests
	17D Naive vs. Control Naïve	<0.0001	non linear regresion curve fit, extra sum-of-squares F tests
	Contralateral, 17D Immunized vs. 17D Naive	<0.0001	non linear regresion curve fit, extra sum-of-squares F tests
	Contralateral, Control Immunized vs. 17D Naive	<0.0001	non linear regresion curve fit, extra sum-of-squares F tests
	Contralateral, Control Immunized vs. Control Naive	0.1968	non linear regresion curve fit, extra sum-of-squares F tests
	Contralateral, 17D Immunized vs. Control Immunized	<0.0001	non linear regresion curve fit, extra sum-of-squares F tests
	Contralateral, 17D Naive vs. Control Naïve	<0.0001	non linear regresion curve fit, extra sum-of-squares F tests
**6D**	17D Immunized vs. 17D Naive	0.0403	Log-rank (Mantel-Cox) test
	17D Naive vs. Control Naïve	0.1609	Log-rank (Mantel-Cox) test
	Control Immunized vs. Control Naive	0.5751	Log-rank (Mantel-Cox) test
	17D Immunized vs. Control Immunized	0.0022	Log-rank (Mantel-Cox) test

**7B**	17D + CD4 vs 17D + CD8	0.0005	Log-rank (Mantel-Cox) test
	17D + CD4 vs 17D + CD4+CD8	0.0101	Log-rank (Mantel-Cox) test
	17D + CD4 vs 17D + Serum	0.137	Log-rank (Mantel-Cox) test
	17D + CD4 vs 17D, no transferred	0.1055	Log-rank (Mantel-Cox) test
	17D + CD4 vs Control, no transferred	0.1912	Log-rank (Mantel-Cox) test
	17D + CD8 vs 17D + CD4+CD8	0.093	Log-rank (Mantel-Cox) test
	17D + CD8 vs 17D + Serum	0.0189	Log-rank (Mantel-Cox) test
	17D + CD8 vs 17D, no transferred	0.0435	Log-rank (Mantel-Cox) test
	17D + CD8 vs control no transferred	0.0005	Log-rank (Mantel-Cox) test
	17D + CD4+CD8 vs 17D + Serum	0.2183	Log-rank (Mantel-Cox) test
	17D + CD4+CD8 vs 17D, no transferred	0.4271	Log-rank (Mantel-Cox) test
	17D + Serum vs 17D, no transferred	0.8023	Log-rank (Mantel-Cox) test
	17D + Serum vs Control, no transferred	0.0222	Log-rank (Mantel-Cox) test
	17D, no transferred vs Control, no transferred	0.039	Log-rank (Mantel-Cox) test
**7C**	17D + CD4 vs 17D + CD8	<0.0001	non linear regresion curve fit, extra sum-of-squares F tests
	17D + CD8 vs 17D + CD4+CD8	0.4311	non linear regresion curve fit, extra sum-of-squares F tests
	17D + CD8 vs 17D + Serum	<0.0001	non linear regresion curve fit, extra sum-of-squares F tests
	17D + CD8 vs 17D, no transferred	<0.0001	non linear regresion curve fit, extra sum-of-squares F tests
	17D + CD8 vs control no transferred	<0.0001	non linear regresion curve fit, extra sum-of-squares F tests
	17D + CD4 vs 17D + Serum	0.1854	non linear regresion curve fit, extra sum-of-squares F tests
	17D + CD4 vs 17D + CD4+CD8	<0.0001	non linear regresion curve fit, extra sum-of-squares F tests
	17D + CD4 vs 17D, no transferred	0.3573	non linear regresion curve fit, extra sum-of-squares F tests

	17D + CD4+CD8 vs Control, no transferred	<0.0001	non linear regresion curve fit, extra sum-of-squares F tests
	17D + CD4 vs Control, no transferred	0.1389	non linear regresion curve fit, extra sum-of-squares F tests
	17D + CD4+CD8 vs 17D + Serum	0.0011	non linear regresion curve fit, extra sum-of-squares F tests
	17D + CD4+CD8 vs 17D, no transferred	<0.0001	non linear regresion curve fit, extra sum-of-squares F tests
	17D, no transferred vs Control, no transferred	0.0053	non linear regresion curve fit, extra sum-of-squares F tests
	17D + Serum vs Control, no transferred	0.0014	non linear regresion curve fit, extra sum-of-squares F tests
	17D + Serum vs 17D, no transferred	0.0508	non linear regresion curve fit, extra sum-of-squares F tests
**7D**	17D + CD4 vs 17D + CD8	<0.0001	non linear regresion curve fit, extra sum-of-squares F tests
	17D + CD8 vs 17D + CD4+CD8	<0.0001	non linear regresion curve fit, extra sum-of-squares F tests
	17D + CD8 vs 17D + Serum	<0.0001	non linear regresion curve fit, extra sum-of-squares F tests
	17D + CD8 vs 17D, no transferred	0.0017	non linear regresion curve fit, extra sum-of-squares F tests
	17D + CD8 vs control no transferred	<0.0001	non linear regresion curve fit, extra sum-of-squares F tests
	17D + CD4 vs 17D + CD4+CD8	0.0788	non linear regresion curve fit, extra sum-of-squares F tests
	17D + CD4 vs 17D, no transferred	0.1421	non linear regresion curve fit, extra sum-of-squares F tests
	17D + CD4 vs Control, no transferred	0.4543	non linear regresion curve fit, extra sum-of-squares F tests
	17D + CD4+CD8 vs 17D + Serum	0.5483	non linear regresion curve fit, extra sum-of-squares F tests

	17D + CD4+CD8 vs 17D, no transferred	0.0007	non linear regresion curve fit, extra sum-of-squares F tests
	17D + CD4+CD8 vs Control, no transferred	0.0003	non linear regresion curve fit, extra sum-of-squares F tests
	17D + CD4 vs 17D + Serum	0.1624	non linear regresion curve fit, extra sum-of-squares F tests
	17D, no transferred vs Control, no transferred	0.6416	non linear regresion curve fit, extra sum-of-squares F tests
	17D + Serum vs Control, no transferred	0.0003	non linear regresion curve fit, extra sum-of-squares F tests
	17D + Serum vs 17D, no transferred	0.0008	non linear regresion curve fit, extra sum-of-squares F tests
	17D + CD4 vs 17D + CD4+CD8	<0.0001	non linear regresion curve fit, extra sum-of-squares F tests
**8B**	Tumor volumes	<0.0001	non linear regresion curve fit, extra sum-of-squares F tests
	survival	0.0046	Log-rank (Mantel-Cox) test
**8C**	17D Chicken eggs vs 17D Vero	0.1018	non linear regresion curve fit, extra sum-of-squares F tests
	17D Chicken eggs vs Control	0.0017	non linear regresion curve fit, extra sum-of-squares F tests
	17D Vero vs Control	<0.0001	non linear regresion curve fit, extra sum-of-squares F tests
**8D**	Control+RIgG vs17D+αIFNAR, tumor volumes	<0.0001	non linear regresion curve fit, extra sum-of-squares F tests
	17D+RIgG vs17D+αIFNAR, tumor volumes	<0.0001	non linear regresion curve fit, extra sum-of-squares F tests
	Control+RIgG vs17D+RIgG, tumor volumes	<0.0001	non linear regresion curve fit, extra sum-of-squares F tests
	Control+RIgG vs17D+αIFNAR, survival	0.9603	Log-rank (Mantel-Cox) test
	17D+RIgG vs17D+αIFNAR, survival	0.0029	Log-rank (Mantel-Cox) test

	Control+RIgG vs17D+RIgG, survival	0.0014	Log-rank (Mantel-Cox) test
**EV1**	UV-17D vs Control	0.692	non linear regresion curve fit, extra sum-of-squares F tests
	UV-17D vs 17D	0.0003	non linear regresion curve fit, extra sum-of-squares F tests
	control vs 17D	<0.0001	non linear regresion curve fit, extra sum-of-squares F tests
**EV3 B**	17D+αGR1 vs 17D+RIgG	0.0576	non linear regresion curve fit, extra sum-of-squares F tests
	17D+αNK1.1 vs 17D+RIgG	0.6569	non linear regresion curve fit, extra sum-of-squares F tests
	17D+RIgG vs Control+RIgG	0.002	non linear regresion curve fit, extra sum-of-squares F tests
	17D+αGR1 vs 17D+RIgG	0.6056	non linear regresion curve fit, extra sum-of-squares F tests
	17D+αNK1.1 vs 17D+RIgG	0.1627	non linear regresion curve fit, extra sum-of-squares F tests
	17D+RIgG vs Control+RIgG	<0.0001	non linear regresion curve fit, extra sum-of-squares F tests
**EV3D**	CD4 depletion	0.0045	Unpaired T test
	CD8 depletion	0.0002	Unpaired T test
	NK depletion	0.0018	Unpaired T test
	Gr1 depletion	0.007	Unpaired T test
**EV4A**	CD8	0.0047	Mann-Whithey test, Two-tailed
	NNK	0.0023	Mann-Whithey test, Two-tailed
	CD4 Tconv	0.0023	Mann-Whithey test, Two-tailed
**EV4B**	CD25+FOXP3+CD4+	0.0734	Mann-Whithey test, Two-tailed
	CD8/Treg	0.5338	Mann-Whithey test, Two-tailed
	Tconv/Treg	0.0023	Mann-Whithey test, Two-tailed
	CD137+CD8+	0.0012	Mann-Whithey test, Two-tailed

	PD-1+CD8+	0.035	Mann-Whithey test, Two-tailed
	CD137+CD4+	0.0082	Mann-Whithey test, Two-tailed
	PD-1+CD4+	0.0012	Mann-Whithey test, Two-tailed
**EV5B**	CD4+CD45.2+, 17D Immunized vs 17D naïve	0.1236	Kruskal-Wallis test + Dunn's multiple comparisons correction
	CD4+CD45.2+, 17D Immunized vs Control Immunized	0.0045	Kruskal-Wallis test + Dunn's multiple comparisons correction
	CD4+CD45.2+, 17D Naïve vs Control Immunized	>0.9999	Kruskal-Wallis test + Dunn's multiple comparisons correction
	CD4+CD45.2+, 17D Immunized vs 17D naïve, Contralateral	>0.9999	Kruskal-Wallis test + Dunn's multiple comparisons correction
	CD4+CD45.2+, 17D Immunized vs Control Immunized, Contralateral	>0.9999	Kruskal-Wallis test + Dunn's multiple comparisons correction
	CD4+CD45.2+, 17D Naïve vs Control Immunized, Contralateral	>0.9999	Kruskal-Wallis test + Dunn's multiple comparisons correction
**EV5C**	CD8+CD45.2+, 17D Immunized vs 17D naïve	0.4917	Kruskal-Wallis test + Dunn's multiple comparisons correction
	CD8+CD45.2+, 17D Immunized vs Control Immunized	0.0035	Kruskal-Wallis test + Dunn's multiple comparisons correction
	CD8+CD45.2+, 17D Naïve vs Control Immunized	0.2998	Kruskal-Wallis test + Dunn's multiple comparisons correction
	PD-1+CD8+CD45.2+, 17D Immunized vs 17D naïve	>0.9999	Kruskal-Wallis test + Dunn's multiple comparisons correction
	PD-1+CD8+CD45.2+, 17D Immunized vs Control Immunized	0.0212	Kruskal-Wallis test + Dunn's multiple comparisons correction
	PD-1+CD8+CD45.2+, 17D Naïve vs Control Immunized	0.1042	Kruskal-Wallis test + Dunn's multiple comparisons correction
	PD-1 MFI in CD8+CD45.2+, 17D Immunized vs 17D naïve	>0.9999	Kruskal-Wallis test + Dunn's multiple comparisons correction
	PD-1 MFI in PD-1+CD8+CD45.2+, 17D Immunized vs Control Immunized	0.0079	Kruskal-Wallis test + Dunn's multiple comparisons correction

	PD-1 MFI in PD-1+CD8+CD45.2+, 17D Naïve vs Control Immunized	0.0736	Kruskal-Wallis test + Dunn's multiple comparisons correction
	CD8+CD45.1+, 17D Immunized vs 17D naïve	>0.9999	Kruskal-Wallis test + Dunn's multiple comparisons correction
	CD8+CD45.1+, 17D Immunized vs Control Immunized	0.0108	Kruskal-Wallis test + Dunn's multiple comparisons correction
	CD8+CD45.1+, 17D Naïve vs Control Immunized	0.1273	Kruskal-Wallis test + Dunn's multiple comparisons correction
	PD-1+CD8+CD45.1+, 17D Immunized vs 17D naïve	0.1775	Kruskal-Wallis test + Dunn's multiple comparisons correction
	PD-1+CD8+CD45.1+, 17D Immunized vs Control Immunized	0.0126	Kruskal-Wallis test + Dunn's multiple comparisons correction
	PD-1+CD8+CD45.1+, 17D Naïve vs Control Immunized	>0.9999	Kruskal-Wallis test + Dunn's multiple comparisons correction
	PD-1 MFI in CD8+CD45.1+, 17D Immunized vs 17D naïve	>0.9999	Kruskal-Wallis test + Dunn's multiple comparisons correction
	PD-1 MFI in PD-1+CD8+CD45.1+, 17D Immunized vs Control Immunized	0.0079	Kruskal-Wallis test + Dunn's multiple comparisons correction
	PD-1 MFI in PD-1+CD8+CD45.1+, 17D Naïve vs Control Immunized	0.0736	Kruskal-Wallis test + Dunn's multiple comparisons correction
**EV5D**	CD8+CD45.2+, 17D Immunized vs 17D naïve	0.8787	Kruskal-Wallis test + Dunn's multiple comparisons correction
	CD8+CD45.2+, 17D Immunized vs Control Immunized	0.5342	Kruskal-Wallis test + Dunn's multiple comparisons correction
	CD8+CD45.2+, 17D Naïve vs Control Immunized	>0.9999	Kruskal-Wallis test + Dunn's multiple comparisons correction
	PD-1+CD8+CD45.2+, 17D Immunized vs 17D naïve	0.5204	Kruskal-Wallis test + Dunn's multiple comparisons correction
	PD-1+CD8+CD45.2+, 17D Immunized vs Control Immunized	0.0136	Kruskal-Wallis test + Dunn's multiple comparisons correction
	PD-1+CD8+CD45.2+, 17D Naïve vs Control Immunized	0.5924	Kruskal-Wallis test + Dunn's multiple comparisons correction

PD-1 MFI in CD8+CD45.2+, 17D Immunized vs 17D naïve	>0.9999	Kruskal-Wallis test + Dunn's multiple comparisons correction
PD-1 MFI in PD-1+CD8+CD45.2+, 17D Immunized vs Control Immunized	0.0077	Kruskal-Wallis test + Dunn's multiple comparisons correction
PD-1 MFI in PD-1+CD8+CD45.2+, 17D Naïve vs Control Immunized	0.114	Kruskal-Wallis test + Dunn's multiple comparisons correction
CD8+CD45.1+, 17D Immunized vs 17D naïve	0.6594	Kruskal-Wallis test + Dunn's multiple comparisons correction
CD8+CD45.1+, 17D Immunized vs Control Immunized	0.5265	Kruskal-Wallis test + Dunn's multiple comparisons correction
CD8+CD45.1+, 17D Naïve vs Control Immunized	>0.9999	Kruskal-Wallis test + Dunn's multiple comparisons correction
PD-1+CD8+CD45.1+, 17D Immunized vs 17D naïve	>0.9999	Kruskal-Wallis test + Dunn's multiple comparisons correction
PD-1+CD8+CD45.1+, 17D Immunized vs Control Immunized	>0.9999	Kruskal-Wallis test + Dunn's multiple comparisons correction
PD-1+CD8+CD45.1+, 17D Naïve vs Control Immunized	0.7944	Kruskal-Wallis test + Dunn's multiple comparisons correction
PD-1 MFI in CD8+CD45.1+, 17D Immunized vs 17D naïve	0.2039	Kruskal-Wallis test + Dunn's multiple comparisons correction
PD-1 MFI in PD-1+CD8+CD45.1+, 17D Immunized vs Control Immunized	0.0108	Kruskal-Wallis test + Dunn's multiple comparisons correction
PD-1 MFI in PD-1+CD8+CD45.1+, 17D Naïve vs Control Immunized	>0.9999	Kruskal-Wallis test + Dunn's multiple comparisons correction
