# Supplementary material for: Repurposing the yellow fever vaccine for intratumoral immunotherapy
Source: EMBO Mol Med. 2019 Nov 19;12(1):e10375. doi: 10.15252/emmm.201910375 (PMC6949490; doi:10.15252/emmm.201910375)
Supplement: Supplementary file 2 — Expanded View Figures PDF [file EMMM-12-e10375-s002.pdf]

# Expanded View Figures

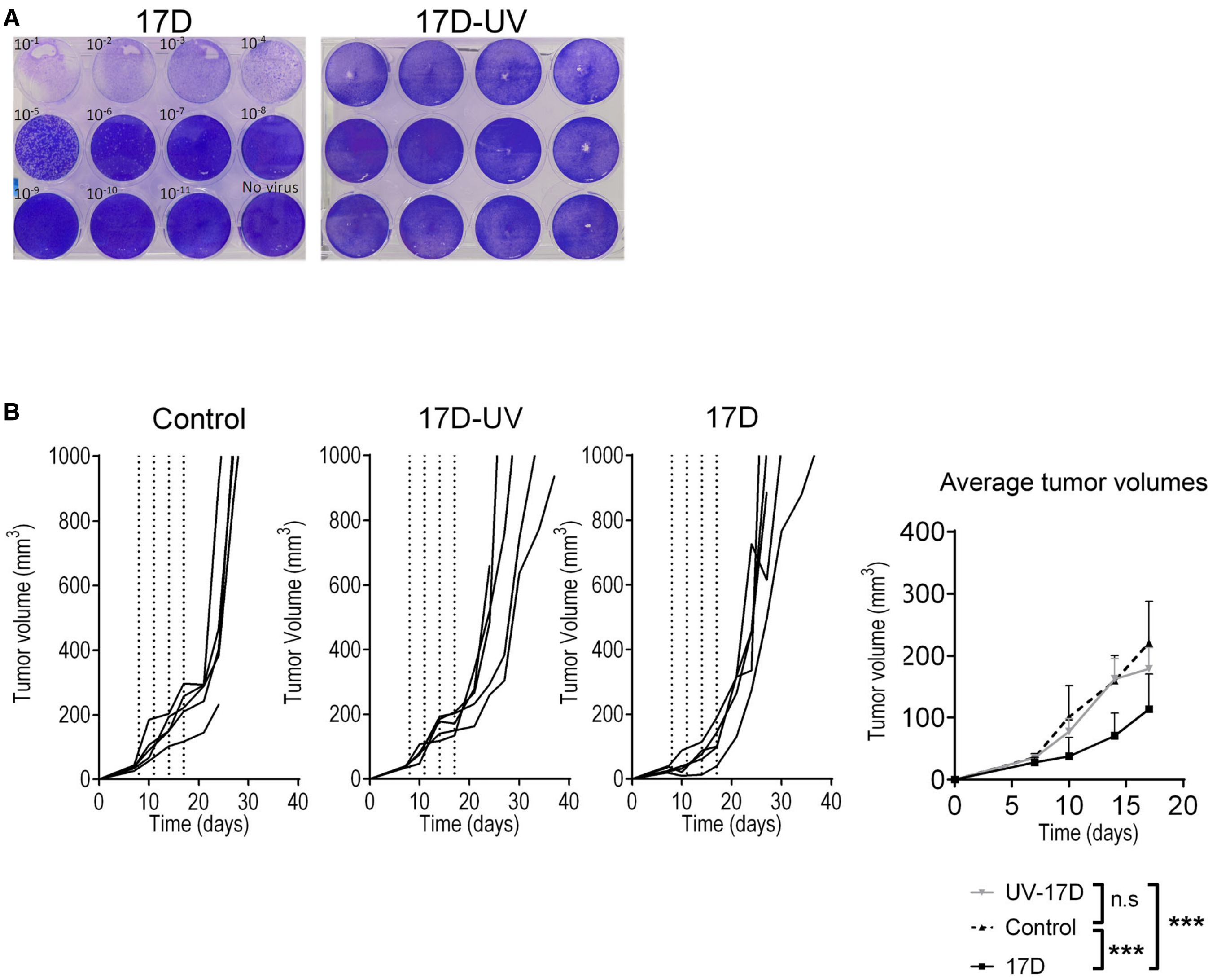


**Figure EV1. Ultraviolet light inactivated 17D virus does not exert antitumor effects.**

A UV exposure inactivates 17D virus infectivity as observed in plaque-forming assays on Vero cells stained with crystal violet.

B Comparative antitumor effect against established MC38-derived tumors intratumorally injected with replication-competent and UV-inactivated 17D virus as indicated. Individual tumor growth follow-up and mean ± SD of tumor lesions are shown, ($n = 5$ per group). Mean tumor volume growth over time was fitted using non-linear regression curve fit. Treatments were compared using the extra sum-of-squares $F$-test. ***$P < 0.001$.


© 2019 The Authors

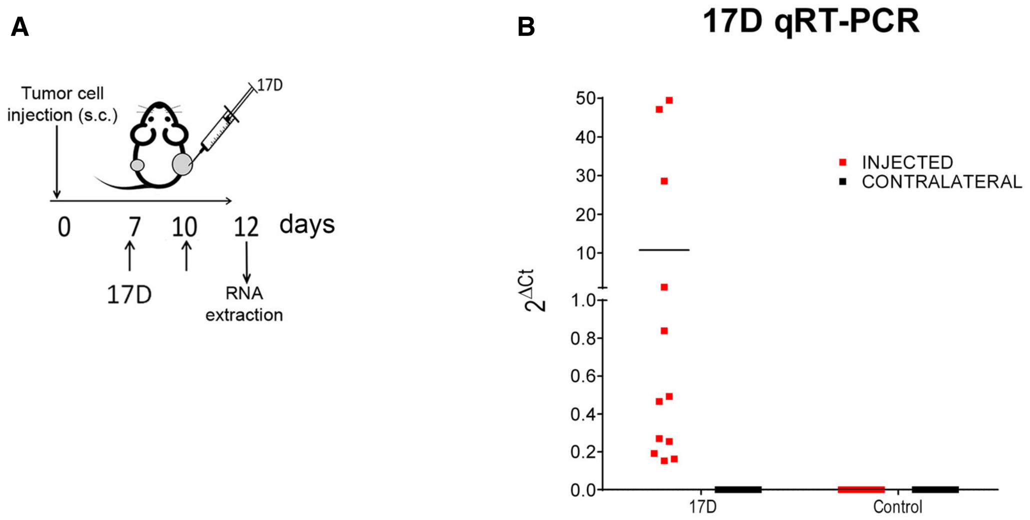


**Figure EV2. Lack of 17D virus detection in contralateral tumors.**

A Schematic representation of the experiment in mice bearing bilateral MC38 tumors that were excised on day +12 (2 days following a second dose of intratumoral 17D virus or control vehicle, $n$ = 9 per group).

B Quantitative RT–PCR detection of 17D in the indicated directly injected or contralateral tumors. Individual cases are represented and the median indicated by a horizontal line.

**Figure EV3. NK1.1 and GR-1 depletions and validation of selective subset depletion experiments.**

A Scheme of depletion experiments as in Fig 4 but depleting NK1.1$^+$ cells (NK and NKT cells) or GR1$^+$ cells (MDSC and granulocytes).

B, C Tumor size follow-up of the indicated experimental groups ($n$ = 6 per group) measuring directly 17D virus-injected (B) and contralateral untreated tumors (C), respectively.

D Verification by flow cytometry of cell depletions in peripheral blood in experiments (mean ± SD, $n$ = 4 per group) from Figs 4 and EV3.

Data information: Mean tumor volume growth over time was fitted using non-linear regression curve fit. Treatments were compared using the extra sum-of-squares $F$-test. Unpaired $T$ test was used to calculate depletion efficiency. $^{**}P < 0.01$$^{***}P < 0.001$.

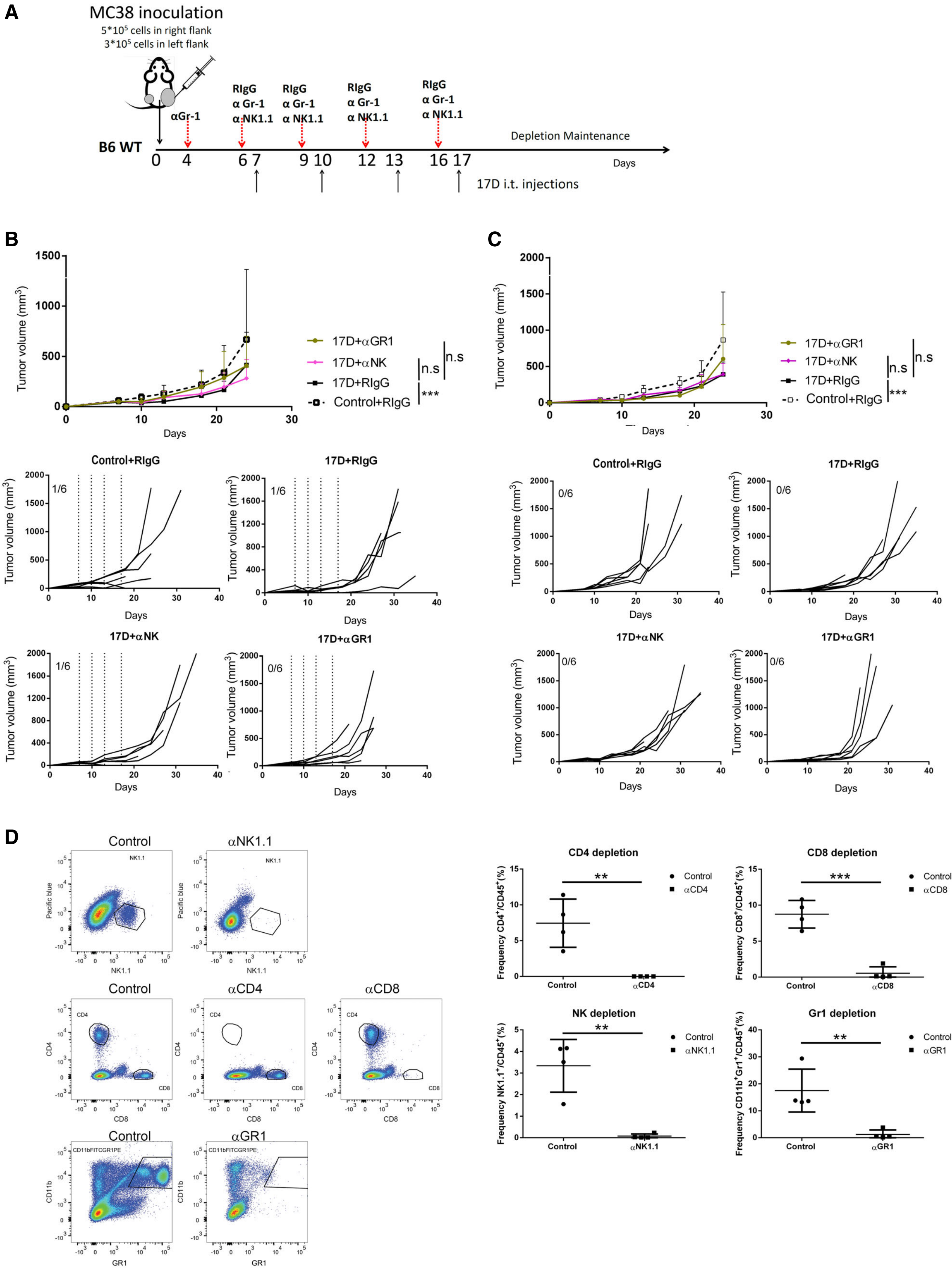


**Figure EV3.**

 © 2019 The Authors

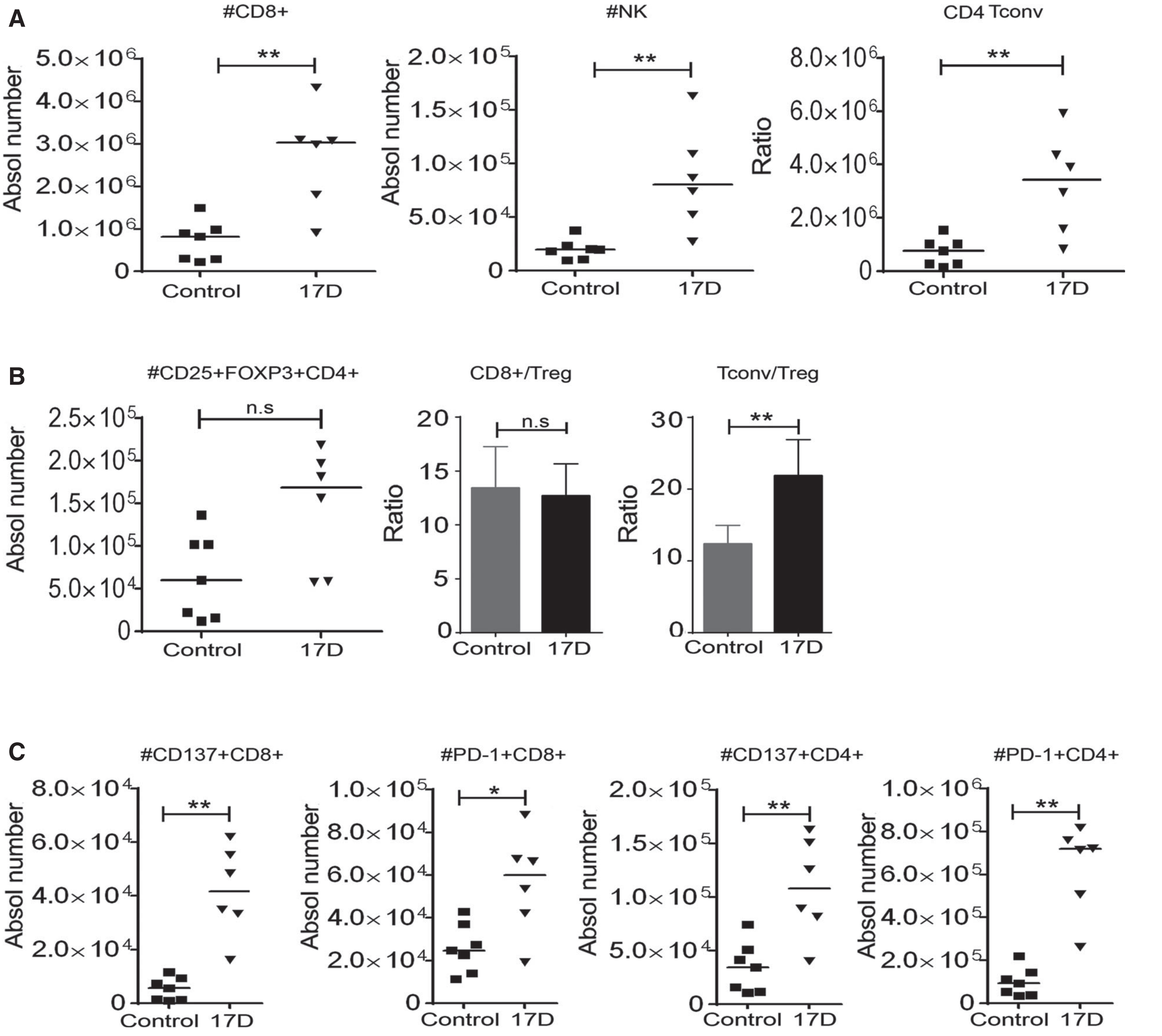


**Figure EV4. T- and NK cell contents in tumor-draining lymph nodes from tumors treated with 17D virus.**

A–C In experiments from Fig 7, (A) absolute numbers of the indicated lymphocyte subsets in tumor-draining lymph nodes. (B) Absolute numbers of Tregs and the corresponding ratios to conventional CD4 and CD8 T cells. (C) Absolute numbers of CD8 and CD4 T cells expressing surface CD137 or PD-1 as indicated ($n$ = 5 for the 17D-injected group and $n$ = 7 for the control group). Mann-Whitney test (two-tailed) was used to evaluate the differences between groups. **$P < 0.01$, *$P < 0.05$, ns: non-significant.

**Figure EV5. Effects of 17D preimmunization in tumor infiltrate after intratumoral injection of 17D.**

A Scheme representing the experiments in which mice were subcutaneously vaccinated with 17D virus 14 days prior to receive MC38 tumor cells in both flanks. On day 6 after tumor inoculation, mice received CD8$^{+}$ splenocytes from a naïve OT-I CD45.1 donor and treated when indicated with intratumoral doses of 17D or control vehicle only given to one of the lesions. On day +13, single-cell suspensions were derived from the excised treated and contralateral tumor nodules. Cell suspensions were processed for multicolor flow cytometry assessments.

B FACS quantification of CD4 T cells per gram of tumor tissue in the indicated groups of mice.

C, D Endogenous (CD45.2) and transferred (CD45.1) OT-1 CD8 cells in injected tumors (C) and contralateral tumors (D).

Data information: Data are shown as individual mice represented by dots and bars depicting mean ± SEM. Kruskal-Wallis test with Dunn's multiple comparisons correction was used for statistics analysis. Immunized+control vehicle $n$ = 7, Immunized+17D $n$ = 6, Naïve+17D $n$ = 5. *$P < 0.05$, **$P < 0.01$, ns: non-significant.

© 2019 The Authors

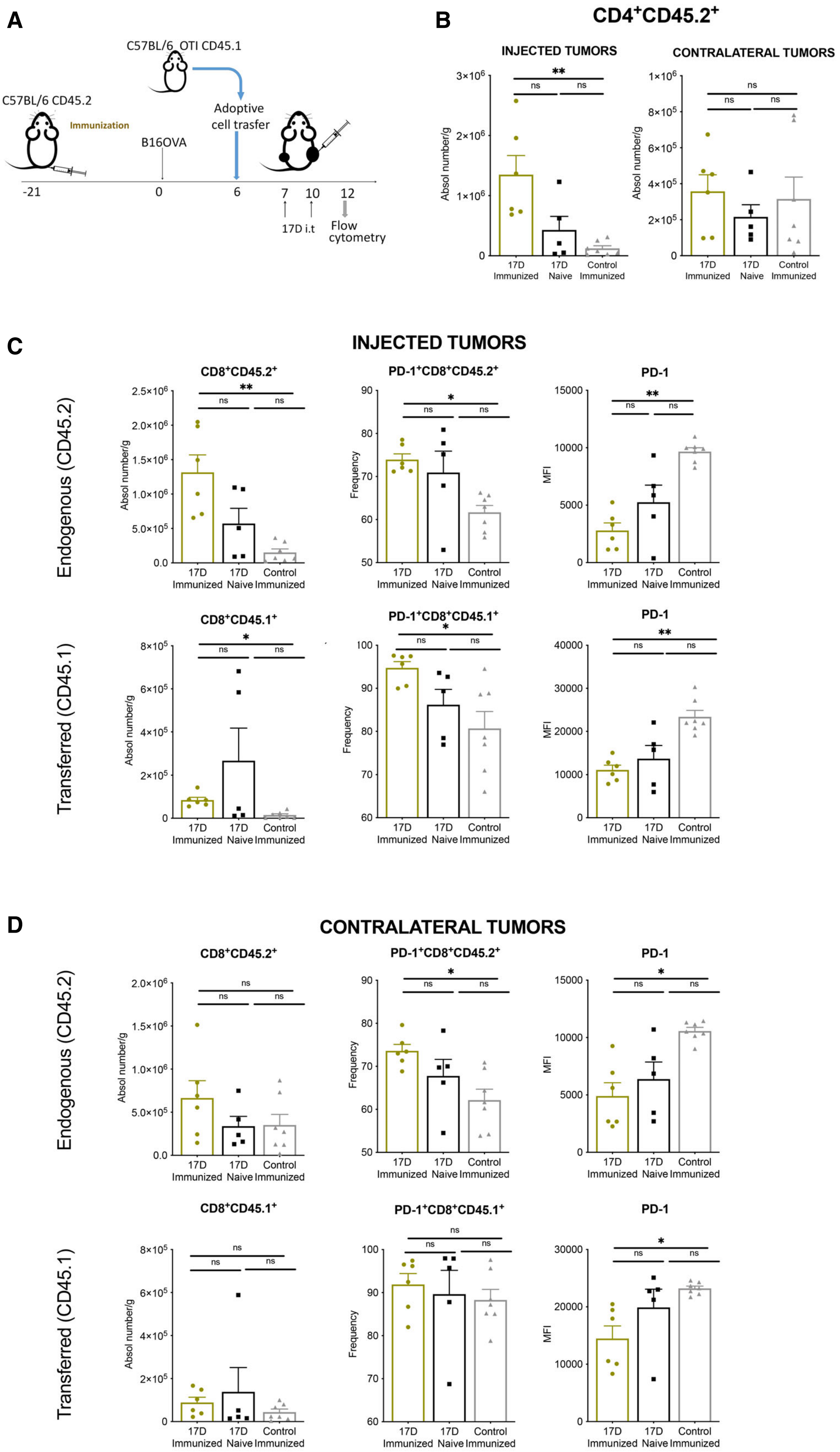


**Figure EV5.**

 © 2019 The Authors
